# Internal Forces Analysis of Prestressed Concrete Box Girder Bridge by Using Structural Stressing State Theory

**DOI:** 10.3390/ma14164671

**Published:** 2021-08-19

**Authors:** Shuo Liu, Yi Zhang, Jun Shi, Baisong Yang

**Affiliations:** 1 School of Civil Engineering, Central South University, Changsha 410075, China; 2019221009@chd.edu.cn; 2 School of Highway, Chang’an University, Xi’an 710064, China; 3 Academy of Combat Support, Rocket Force University of Engineering, Xi’an 710025, China; zhangyi_lijiacun@163.com; 4 National Engineering Laboratory for High Speed Railway Construction, Changsha 410075, China; 5 Key Lab of Structures Dynamic Behavior and Control of the Ministry of Education, Harbin Institute of Technology, Harbin 150090, China; 18S133169@stu.hit.edu.cn

**Keywords:** stressing state, mutation, numerical shape function, large cantilever box girder bridge, characteristic load

## Abstract

This paper analyzes the working behavior characteristics of a prestressed concrete transverse large cantilever continuous (PCTLCC) box girder bridge model based on structural stressing state theory and the numerical shape function (NSF) method. At first, the normalized generalized strain energy density (GSED) is established to model the stressing state of the bridge model. Subsequently, the Mann Kendall (M–K) criterion is applied to detect three characteristic loads, respectively, elastic–plastic branch load *P* (200 kN), failure load *Q* (300 kN), and progressive failure load *H* (340 kN), and the failure load *Q* is found to be the starting load of the damage process of the bridge model, rather than the ultimate load where the structure has been destroyed. Finally, the NSF method is adopted to interpolate the test data, and a detailed analysis for the variation characteristics of the working behavior of the bridge model under loads is performed based on the interpolation results. The characteristic load detection method and experimental data extension method for PCTLCC box girder bridge established in this study can provide valuable references for the design and analysis of such bridges.

## 1. Introduction

Prestressed concrete transverse large cantilever continuous (PCTLCC) box girder bridges are widely constructed for their rational working performance, outstanding span adaptability, and structural economy to meet the requirements of modern infrastructure in traffic conditions [1,2,3]. The box girder section can effectively lighten the self-weight of the structures and provide high torsional rigidity to resist the torsion under eccentric load [4,5]. In addition, prestress can not only enhance the spanning capacity of bridges but also restrain the development of cracks and improve the durability of bridges [6]. At the same time, the cantilever structure can reduce the width of piers, increase the clearance under the bridge, and achieve the purpose of saving engineering. Although the PCTLCC box girder bridge combines the advantages above, they bring researchers and engineers the difficulty of analysis and design as well.

The structural working behavior of prestressed concrete box girder bridges is relatively complicated due to the prestress effect as well as different loading cases. Therefore, numerous scholars have carried out considerable experiments and simulations on prestressed concrete box girder bridges to study the unascertained working characteristics of this type of bridge and contributed to their application in engineering. Yu et al. [7] analyzed the spatial stress of long-span prestressed concrete box girder bridges during the construction stage and service stage and concluded that the main tensile stress can be calculated more accurately by using shell cells than beam ones. Zhang et al. [8] discovered that the initial casting temperature of concrete is the most significant parameter that controls cracking of prestressed concrete box girders on pier tops at the cement hydration age and increasing the curing temperature is detrimental to prevent cracking. Based on the genetic optimization algorithm, Huang et al. [9] found that creep and prestress loss have a significant effect on the vertical deflection of the main and side spans. Moreover, the prestress loss can change the ratio of deflection of main and side spans. Yang et al. [10] studied the analysis method of web cracking probability of prestressed concrete box girder bridges and pointed out that compared with the traditional prestressing tensioning technique, the doubled tensioning technique is significantly more effective in reducing the web cracking probability. Undoubtedly, these research results greatly promoted the application of prestressed concrete box girder bridges in engineering projects.

Due to the special structure of the prestressed concrete cantilever bridge, its internal force distribution and mechanical performance are often more complex than the ordinary box girder bridge. Hence, a lot of studies have been carried out to explore the unknown working performance of the prestressed concrete cantilever bridge. Yu et al. [11] analyzed a prestressed concrete box girder bridge with large cantilevered wings, concluding that the distributions of the stress and cracks under the load case of asymmetrical load were asymmetrical and cracks were more likely to appear in the root of the cantilever wing comparing with the symmetrical load. Chen [12] discovered that transverse prestressing can effectively reduce the deflection of the section and the transverse tensile stress of the top plate in the box girder, and the smaller the spacing of the transverse prestressing bars, the more obvious the reduction effect is. Nie [13] performed finite element simulations for a PCTLCC box girder bridge and found that the presence of transverse prestress has little effect on the longitudinal bridge forces of the box girder but has a significant effect on the transverse stresses in the deck slab. In addition, some researchers conducted experimental studies on the cracking resistance of the wide-box girder bridge with a large cantilever [14,15].

To sum up, the existing research can greatly promote the development and application of the PCTLCC box girder bridge indeed, while there still exist some problems puzzling researchers, which limits the development of this kind of bridge to some degree. Due to the complex stressing state of the bridge, various formulas based on different assumptions and preconditions have been used to calculate the bearing capacity, and these calculation results are often conservative and diverse from each other, leading to the overuse and/or irrational use of materials. Moreover, there is little research on its specific stressing state changing characteristics in the process of structural failure, such as the distribution of sectional stresses, internal forces, etc. In addition, the high experimental costs and long test period result in limited experimental data of the PCTLCC box girder bridge, which are insufficient to update the existing theories.

In order to remedy the above research deficiencies, it is necessary to investigate new analytical theories to explore the working performance of the PCTLCC box girder bridge and to develop methods to extend the limited experimental data to further analyze the internal force changes during the damage of the PCTLCC box girder bridge. Specifically, the authors experimentally analyzed the working behavior characteristics of the PCTLCC box girder bridge model based on structural stressing state theory and the numerical shape function (NSF) method, revealing its stressing states’ changing characteristics in the whole loading process. First, the generalized strain energy density (GSED) was creatively modeled to evaluate the structural stressing state mode of the PCTLCC box girder bridge. Subsequently, the Mann–Kendall (M–K) criterion in hydrometeorology was innovatively used to detect characteristic loads and reveal abrupt structural features. Furthermore, based on the NSF method with high accuracy, the limited strain data were expanded to obtain the strain/stress fields and internal forces, so as to further analyze the stressing state characteristics of the bridge model. The structural stressing state theory and NSF method proposed in this study can provide a new approach to the design and analysis of PCTLCC box girder bridges and other types of structures.

## 2. Methods for Modeling and Analyzing Structural Stressing State

### 2.1. Structural Stressing State Concept and GSED Curve

The authors defined the stressing state of a structure as its working behavior, which is characterized by the distribution pattern of strain energy density values, displacements, strains, and stresses of measuring points [16,17]. Generally, the strain energy density *E_i_* of the *i*-th point can be calculated by

(1) $$E_{i}=\int \sigma_{1}d\varepsilon_{1}+\sigma_{2}d\varepsilon_{2}+\sigma_{3}d\varepsilon_{3}$$

where *σ*_1_, *σ*_2_, *σ*_3_ and *ε*_1_, *ε*_2_, *ε*_3_ are three principal stresses and strains, respectively; *E_i_* is the *i*-th strain energy density.

However, it is difficult to measure the stress or strain at one point in three directions in the experiment; thus, this study creatively chose the GSED as the characteristic parameter to express the stressing state at a point, and further analyzed the characteristics of the working behavior of PCTLCC box girder bridge. Consequently, Equation (1) is simplified as

(2) $$E_{ij}^{k}=\int_{0}^{\varepsilon_{ij}^{k}}\sigma (\varepsilon )d\varepsilon$$

where $E_{ij}^{k}$ is the GSED of the *i*-th point for the *j*-th subpart of the structure at the *k*-th load step; *σ*(*ε*) is the constitutive curve of the material at the measuring point; $\varepsilon_{ij}^{k}$ is the measured strain of the *i*-th point in a certain direction for the *j*-th subpart of the structure at the *k*-th load step. Furthermore, then the GSED sum of all groups (sub-parts) for the *j*-th subpart of the structure at *k*-th load step can be calculated by

(3) $$E^{k}=\sum_{k}\sum_{i}E_{ij}^{k}$$

where *E^k^* is the GSED sum of all groups (sub-parts) to express the stressing state of the whole structure at the *k*-th load step. In order to exclude the influence of units, values, and other factors, the normalized GSED sum $E_{\mathrm{norm}}^{k}$ is adopted to characterize the structural stressing state at each load step, which can be calculated by

(4) $$E_{\mathrm{norm}}^{k}=\frac{E^{k}}{E^{\max}}$$

where $E_{\mathrm{norm}}^{k}$ is the normalized GSED sum of the whole structure at the *k*-th load step. Then, it can be seen that the $E_{\mathrm{norm}}^{k}$–*F* curve can vividly exhibit differential structural stressing states and corresponding characteristics.

### 2.2. M–K Criterion

The Mann–Kendall (M–K) is a widely used trend analysis tool currently without the necessity for samples to comply with certain distributions or interference of a few outliners [18,19,20]. In this study, the M–K criterion was found to be applicable to the analysis of *E*′–*F* curves; thus, it was innovatively applied to the characterization of *E*′–*F* curves to distinguish the stressing state leap of the PCTLCC box girder bridge. Furthermore, it was assumed that the sequence of {$E_{\mathrm{norm}}^{k}$} (the load step *k* = 1, 2, …, *n*) is statistically independent. Then, a new stochastic variable *b_k_* at the *k*-th load step is defined as

(5) $$b_{k}=\sum_{l}^{k}h_{l}\left(2\leq k\leq n\right),\;h_{l}=\left\{ \begin{array}{l} +1\\ 0 \end{array}\right.\begin{array}{l} E_{\mathrm{norm}}^{l}>E_{\mathrm{norm}}^{m}\left(1\leq m\leq l\right)\\ \mathrm{otherwise} \end{array}$$

where *h_l_* is the cumulative number of the samples; “+1” means adding one more to the existing value if the inequality on the right side is satisfied for the *j*-th comparison.

The mean value *D*(*b_k_*) and variance *Vie*(*b_k_*) of *b_k_* are calculated by

(6) $$D\left(b_{k}\right)=\frac{k\left(k-1\right)}{4}\left(2\leq k\leq n\right)$$

(7) $$Vie\left(b_{k}\right)=\frac{k\left(k-1\right)\left(2k+5\right)}{72}\left(2\leq k\leq n\right)$$

Under the assumption that the {$E_{\mathrm{norm}}^{k}$} sequence is statistically independent, a new statistic *NF*_k_ is defined by

(8) $$NF_{k}=\left\{ \begin{array}{l} 0\\ \frac{b_{k}-D\left(b_{k}\right)}{\sqrt{Vie\left(b_{k}\right)}} \end{array}\right.\begin{array}{l} k=1\\ 2\leq k\leq n \end{array}$$

Thus, all the *NF_k_* data can form an *NF_k_*–*F* curve. Applying the same approach to the inverse {${E^{\prime }}_{\mathrm{norm}}^{k}$} sequence, the *NB_k_*–*F* curve can be obtained. Consequently, the *NF_k_* and *NB_k_* curves can intersect at the mutation point of the $E_{\mathrm{norm}}^{k}$–*F* curve, which is taken as a criterion for distinguishing structural stressing state leaps. The M–K method has a high recognition rate for mutations, and its discriminatory results are not greatly affected when the data fluctuates.

### 2.3. Method for Expanding Experiment Data

Experimental data are often so limited due to the cost and period limitations that some mechanisms and characteristics of the structure cannot be discovered. Therefore, a reasonable spatial interpolation method is needed to extend the experimental strain data to obtain more hidden laws. The numerical shape function (NSF) interpolation method was developed to expand the limited experimental data of the bridge model to overcome the shortcoming of conventional interpolation methods that cannot make accurate interpolation of experimental data for structural analysis [21]. Compared with other interpolation methods, the NSF method has higher interpolation accuracy and clearer physical meaning. Applying this method, the changing characteristics of the stressing state of the PCTLCC box girder bridge could be further investigated.

The NSF method was developed from the shape function in the finite element method (FEM). The element displacement field in FEM is expressed as a linear combination of shape functions, while the node displacement is exactly the weight of the corresponding shape function. For a rectangular element with four nodes in the regular coordinate system (*ξ*, *η*), the element displacement field can be expressed as

(9) $$U=\sum_{i=1}^{4}u_{i}N_{i}(\xi ,\eta )$$

where *U* is the displacement field of the rectangular element; *N_i_* is the shape function of node *i* where the node has a displacement *u_i_*.

However, the displacement field of each element is not accurate enough, but according to the principle of FEM, the simulated displacements of a finite element model assembled with enough small elements can reflect the deformation characteristics of the plate with high order continuity through the equilibrium equation established by the principle of virtual displacement. Thus, by dividing the interpolation space into small enough units and using finite element software, a more reasonable shape function can be simulated. Therefore, it can be called a numerical shape function, which could be used to perform a more precise and meaningful interpolation process.

**Figure 1:**
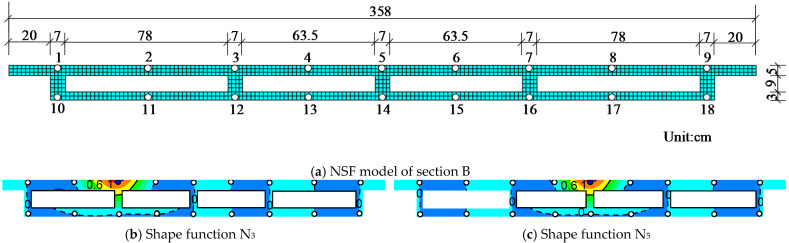
NSF model and contour map of the numerical shape function: (**a**) NSF model of section B; (**b**) shape function *N*_3_; (**c**) shape function *N*_5_.

Like the conventional shape function, the numerical shape functions of several measuring points can be obtained through the numerical simulation of the specific model. Taking the PCTLCC box girder bridge in this study as an example, the experimental data of 18 measuring points of the cross-section are extended. Figure 1a shows the element divisions of the plate model with the size of 20 × 20 mm^2^ of each element using the Shell 181 element. Furthermore, the 18 sampled points are the basic nodes of the numerical shape function. Thus, the numerical shape function can be derived by the following procedure:

1. Take the whole plate as a super element and divide it into suitable small elements;
2. For instance, the shape function of measuring point 3 can be obtained by applying a unit displacement at point 3 (blue dot) along the *z*-axis, while other points (white dots) are fixed. Then, static analysis is performed to derive the displacement field *N*_3_, as shown in Figure 1b;
3. Similarly, the numerical shape function of *N*_2_–*N*_18_ can be acquired, in which *N*_5_ is shown in Figure 1c. If the deflections of the measuring points are given, the displacement field of the plate can be calculated by Equation (10):

(10) $$D=\sum_{i=1}^{M}u_{i}N_{i}$$

where D is the displacement field of the plate; $N_{i}=\left[N_{i}\left(x_{1}\right),N_{i}\left(x_{2}\right)\cdots N_{i}\left(x_{j}\right)\cdots N_{i}\left(x_{n}\right)\right]$ is the numerical shape function of measuring point *i*; *N_i_*(*x_j_*) is the function value at element node *x_j_*; and *n* is the total node number of the plate. The measuring node has a displacement *u_i_*, and *M* is the number of measuring points.

Correspondingly, the limited measuring strain on the cross-section can be expanded by applying the NSF method to obtain the strain field, and the expanded stress data can be calculated through the constitutive relation of the material. Consequently, the above extended experimental data can be used to plot the stress–strain field contours and lay the foundation for in-depth analysis of the structural stress state.

### 2.4. Internal Force Calculation of Cross-Section

Under the given loads, the bridge model is mainly subjected to bending moments and axial forces; hence, the sectional internal forces are constructed based on the expanded stress data in order to deeply investigate the working characteristics of the bridge model. Here, the axial force is calculated by summing the product of longitudinal stress and area for each element, and the in/out-plane bending moment is achieved through summing the product of longitudinal stress, vertical/horizontal distances, and area for each element, as shown in Equations (11)–(13):

(11) $$N=\int_{A}\sigma dA=\sum_{A}\sigma_{i}A_{i}$$

(12) $$M^{\mathrm{in}}=\int_{A}\sigma ydA=\sum_{A}\sigma_{i}y_{i}A_{i}$$

(13) $$M^{\mathrm{out}}=\int_{A}\sigma xdA=\sum_{A}\sigma_{i}x_{i}A_{i}$$

where *N* is the axial force of the section, *M*^in^ is the in-plane bending moment of the section, *M*^out^ is the out-plane bending moment of the section, *σ_i_* is the longitudinal stress of the *i*-th element, *A_i_* is the area of the *i*-th element, and *x_i_* and *y_i_* are the horizontal and vertical distances of the *i*-th element from the neutral axis, respectively.

**Figure 2:**
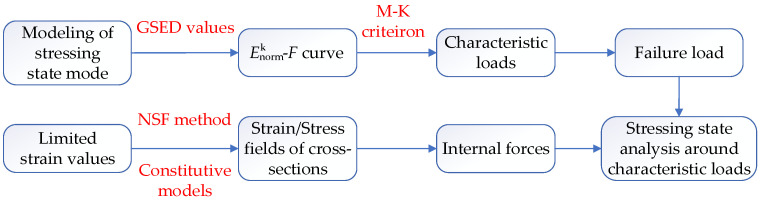
Stressing state analysis idea of the PCTLCC box girder bridge.

Overall, the analytical idea of this study can be clearly demonstrated by Figure 2. First, the method of modelling structural stressing state was used to analyze the working behavior of PCTLCC box girder bridge under loads. The energy-based parameter GSED was calculated, and the $E_{\mathrm{norm}}^{k}$–*F* cure was obtained. Applying the M–K criterion, the characteristic loads were detected. Moreover, due to the limited strain, data could not provide a clear reflection of structural working behavior, so the NSF method was applied to interpolate data at non-sampled locations so as to reflect the stressing state of the bridge model in detail. With the help of test data and material constitutive models, the strain and stress fields of the cross-sections could be obtained for analyzing the stressing state characteristics of the bridge model around the characteristic loads.

## 3. Experiment and Simulation of PCTLCC Box Girder Bridge Model

### 3.1. Experiments on PCTLCC Box Girder Bridge Model

**Figure 3:**
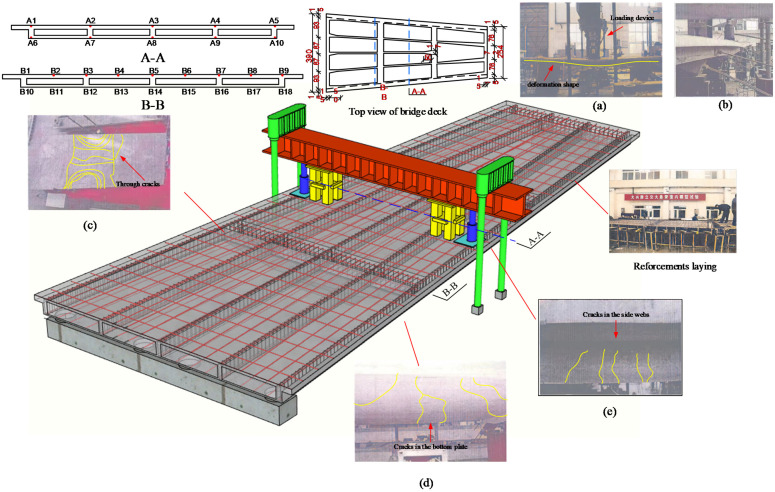
Test loading diagram and test phenomenon: (**a**) loading device; (**b**) deformation state; distribution of cracks in the (**c**) top slab; (**d**) bottom slab in the span; (**e**) side slab in the span.

According to a prestressed concrete continuous box girder bridge with a transverse long cantilever in Xi’an, China, Gao [22] designed and carried out an experiment of a 1/10 scale bridge model with a total span of 9 m, as shown in Figure 3. The bridge model had three spans, each of 3 m, and the first span was a three-cell box girder bridge, while the second and the third spans were four-cell box girder bridges. The diameter of the stressed reinforcements and prestressed steel strands were, respectively, 10 mm and 15.24 mm. The yield strength and elastic modulus of stressed reinforcements were, respectively, 517.8 MPa and 213 GPa, and the ultimate strength and elastic modulus of prestressed steel strand were, respectively, 1860 MPa and 195 GPa. The bridge model was made of C50 concrete, with a standard compressive strength of 40 MPa and an elastic modulus of 40 GPa.

Figure 3 shows the loading device of the experiment. It can be seen that the loading for the bridge model was achieved by the reaction force of the QL32T jacks (dark blue), and the 30 cm × 25 cm rubber pads (light blue) under the jacks were used to convert concentrated load into a surface load of the small area. The loads were applied to the mid-span side webs of the bridge model at fixed increments of 20 kN. The steel I-beam (red) was supported by the steel cushion blocks (yellow) in unloaded phases, and the blocks would gradually separate from the bridge model as the load increased. The reaction frame (green) was fixed on the ground in order to provide a reaction for the steel I-beam. Moreover, the electric resistance strain gauges with the model of BF350 were arranged on the surface of main steel bars at the top and bottom plates, and the distribution of the strain measuring points was symmetrical horizontally and vertically. The strain data were collected via the TZT3826E strain collector produced by Jiangsu Test Equipment Manufacturing Co., LTD (Taizhou, China).

After loading, transverse and longitudinal connecting cracks appeared in the bottom plate, and vertical connecting cracks appeared in the flange plate and web plate. At this time, cracks with different widths appeared on the roof of the model bridge. The deformation state of the bridge model after failure is shown in Figure 3a,b, and it can be seen that the deflection value at the mid-span was the largest. The distribution of cracks throughout the top slab of the bridge model is shown in the Figure 3c, and it can be seen that there were penetration cracks along both the transverse and longitudinal directions. In addition, the distribution of cracks in the bottom and side webs in the span are shown in Figure 3d,e.

### 3.2. FEM Simulation of the PCTLCC Box Girder Bridge Model

**Figure 4:**
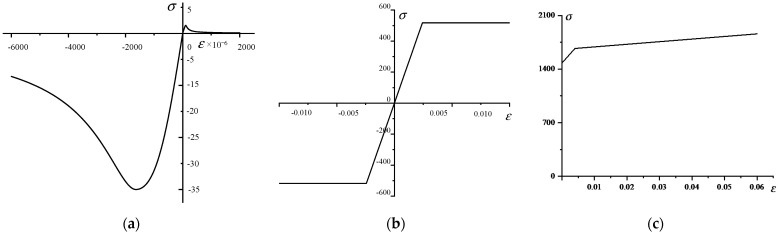
The constitutive models of materials: (**a**) concrete; (**b**) reinforcement; (**c**) prestressed steel strand.

**Figure 5:**
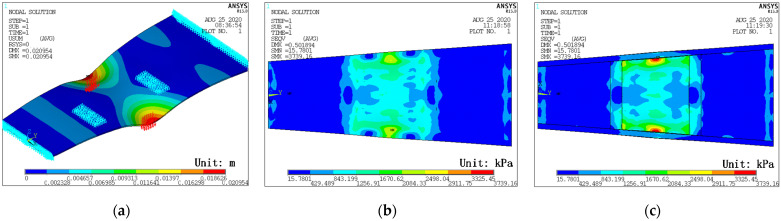
The simulation results of the bridge model: (**a**) displacement contours; (**b**) Von Mises stress contours (top view); (**c**) Von Mises stress contours (bottom view).

ANSYS finite element software was adopted to model the PCTLCC box girder bridge with a top-down modeling approach. Firstly, the beam model was established with three-dimensional CAD software, and the ANSYS program was imported to generate the ANSYS solid model. The solid element type, Solid 65, was selected for mapping mesh generation, and the constitutive models of concrete and reinforcements, respectively, adopted the widely used quadratic parabola plus horizontal straight-line mode suggested by Rusch and the ideal elastoplastic model, as shown in Figure 4a,b [23,24,25]. The constitutive model of the prestressing strand is shown in Figure 4c, and the overall embedded constraint type was used to couple its degrees of freedom to the concrete units at the corresponding locations. Moreover, the initial prestress in the prestressing strand was simulated by means of the cooling method, and the value of cooling was determined according to the value of the initial prestress. Figure 5a shows the load and boundary conditions and the displacement contours under 400 kN, and the Von Mises contours in the top and bottom view are shown in Figure 5b,c. It can be seen that the cloud diagram obtained from the simulation was relatively consistent with the actual test situation, which verifies the accuracy of the model to a certain extent. In addition, Figure 8a in the latter section shows that the strain-*F* curves obtained from the simulation agreed well with the test results; thus, the accuracy of the model could be further validated.

## 4. Stressing State Analysis of the Bridge Model

### 4.1. GSED-Based Stressing State Mode and Characteristic Parameter

**Figure 6:**
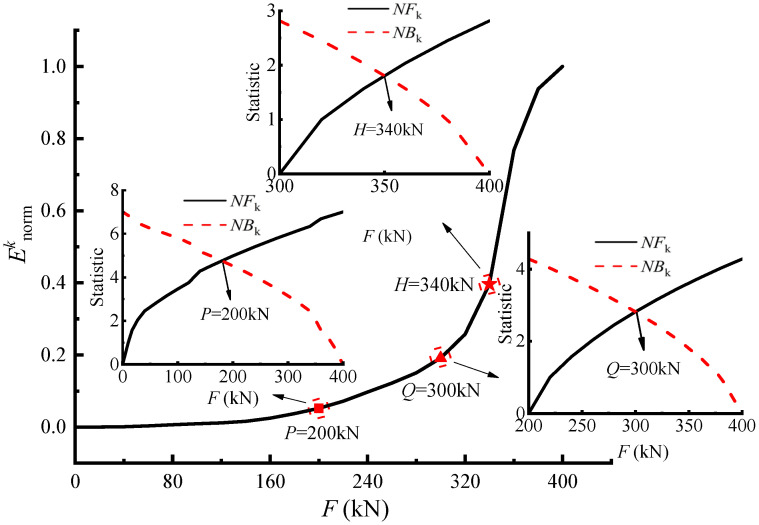
The $E_{\mathrm{norm}}^{k}$–*F* and M–K statistic curves.

In order to reveal the structural stressing state characteristics of the bridge model, the normalized GSED values were chosen as the characteristic parameter to better investigate the developing tendency, as calculated by Equations (2)–(4). Then the $E_{\mathrm{norm}}^{k}$–*F* curve could be plotted to analyze the working behavior of the bridge model. First, based on the overall $E_{\mathrm{norm}}^{k}$–*F* curve, the characteristic load *P* was obtained using the M–K criterion. Then, the curve after the load *P* was analyzed with the M–K criterion to obtain the characteristic load *Q*. Finally, the curve after *Q* was statistically calculated based on the M–K criterion to obtain the characteristic load *H*. Hence, three characteristic loads, respectively, *P* = 200 kN, *Q* = 300 kN, and *H* = 340 kN, were distinguished from the $E_{\mathrm{norm}}^{k}$–*F* curve as shown in Figure 6.

It can be seen that the characteristic parameter $E_{\mathrm{norm}}^{k}$ increased slowly before load *P*, and there were no cracks on the bridge model until 120 kN. Thereafter, small cracks started to appear in the web and bottom plate of the mid-span section, and the width of the cracks was less than 0.05 mm, indicating that before load *P*, the bridge model was in a stable elastic stressing state phase. After that, due to the rapid development of concrete cracks and the reduction of section stiffness, $E_{\mathrm{norm}}^{k}$ increased still relatively slowly but nonlinearly, signifying that the whole bridge model entered the elastic–plastic stressing state phase. From load *Q* on, $E_{\mathrm{norm}}^{k}$ increased sharply and rapidly, showing a different trend than before, which indicates that the bridge model mutated from the stable stressing state to the unstable one, namely the failure stressing state phase. The mutation characteristic of the structural stressing state is its inherent property and revealed according to the natural law from quantitative change to qualitative change of a system, which represents the starting point of the bridge model’s failure process.

As the failure of the bridge model is a process of gradual development, the failure stage also needs to be studied so as to reflect the possible collapse modes of the bridge model. It could be observed that when the load exceeded *H*, $E_{\mathrm{norm}}^{k}$ continued to increase at a higher growth rate than before, owing to further plastic development of the bridge; hence, load *H* is defined as the progressive failure load. In this stage, the number, width, and depth of cracks increased further, eventually forming connecting cracks. There were elliptical arc cracks around the loading points in the mid-span loading area, and the cracks around the two loading points were geometrically symmetrical. The phenomena exposed above are derived from the concept of structural progressive failure. The first and progressive failures of the bridge model further reflect the characteristics of the structural gradual failure process or vividly embody the structural failure evolution.

Overall, the structural stressing state of the bridge model could be divided into three stages by characteristic loads *P* and *Q*:

1. Before load *P*, the bridge is basically in a stable elastic stressing state, and load *P* could be defined as the demarcation point from elastic stressing state to elastic–plastic one;
2. From *P* to *Q*, the bridge model begins to enter the nonlinear stressing state, namely the elastic–plastic stressing state phase;
3. After failure load *Q*, the stressing state of the bridge model changes suddenly and qualitatively. Furthermore, the plastic damage of the bridge model develops further when the load exceeds *H*.

### 4.2. Analysis of Stressing State Modes Based on the Measured Strain

**Figure 7:**
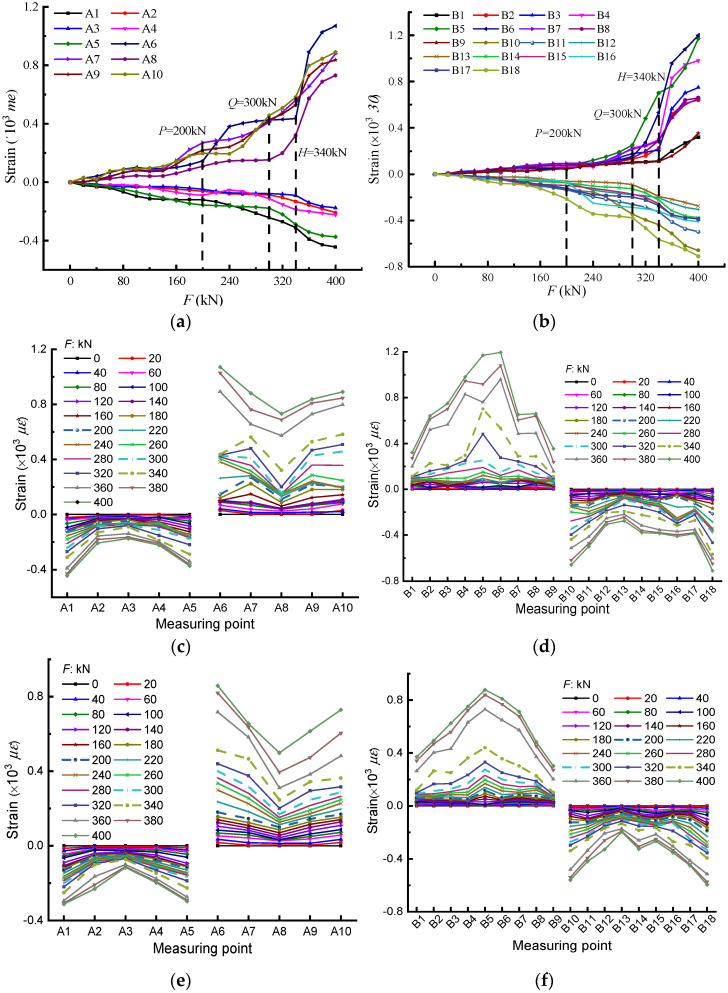
The changing characteristics of strain-based stressing state modes: the trend changing characteristics of strain-based stressing state mode of (**a**) section A; (**b**) section B in the experiment; the distribution pattern changing characteristics of strain-based stressing state modes of (**c**) section A; (**d**) section B in the experiment; (**e**) section A; and (**f**) section B in the simulation.

The strain-*F* curves of all measuring points of section A are plotted in Figure 7a, and it could be observed that all the curves almost kept slow growth before the load *P*, and the strain of each measuring point was relatively close, indicating that the bridge model was in a stable elastic stress state. At this stage, only very small cracks appeared in the web and bottom plate of the mid-span section. After load *P*, the curves began to diverge, and the growth of each curve began to increase in a nonlinear way, embodying inconsistent plastic development at various positions on the cross-section in the elastic–plastic stressing state. At the same time, the cracks kept expanding and extending. When it arrived at load *Q*, the strain curves of several measuring points (especially at A1, A5, and A8) increased sharply, and the strain difference of each measuring point gradually increased, signifying that the bridge model entered the failure stage. In this stage, all the strains of measuring points underwent abrupt changes, revealing the fierce plastic development of the whole bridge model. Moreover, the number of new cracks was increasing, and existing cracks were gradually expanding, accompanied by the formation of penetration cracks (especially in the structure loading area). As for section B shown in Figure 7b, its trend of changing characteristics was similar to that of section A; therefore, the strain-*F* curves also had the mutation characteristics at loads *P*, *Q*, and *H*, which is consistent with that revealed from the characteristic parameter $E_{\mathrm{norm}}^{k}$.

Certainly, the strain–location curves can describe the distribution mode of the strain-based stressing state, as illustrated in Figure 7c,d. The mutation characteristics around the three characteristic loads for the two sections could also be seen in the strain-based mode distribution diagram in shapes and increments, such as the shapes of A6, A7, and A8 for section A and those of B4, B5, B6, and B7 for section B, the increments of A6, B5, B6, etc. Furthermore, it can be seen that before load *P*, the strain at each measuring point on the section was small, the growth rate of strain for each point was relatively slow, and there was no obvious redistribution of stress. Before and after loads *Q* and *H*, the increasing speed of the strain at some measuring points was faster, while the strain at some measuring points was slower, which is a direct reflection of the redistribution of stress and reveals the coordinated work behavior of the bridge model. Furthermore, the strain in section B was small in the middle of the top plate and large in the middle of the soleplate, which indicates that the transverse bending moment of this bridge may make the stress distribution in the width direction of the section uneven. Moreover, after load *H*, the peak characteristics of some points, such as, A1, A6, A8, etc., could reflect their decisive roles in their respective stressing state phases.

Figure 7e,f also shows the strain-*F* curves of all measuring points of section A and section B based on the simulation, it could be found that the maximum values of experimental data exceeded those of the simulated ones. Moreover, comparing Figure 7c with Figure 7e, it can be seen that after load *H*, the strains of A2 and A4 in Figure 7e were much larger than those of A3, while the strains of the three measuring points in Figure 7c were approximately the same. Similarly, the change characteristics of A8, A9, A10, B4, B5, and B6 could be observed as well, while from the whole point of view the mutation characteristics at the characteristic points for simulation data were also very obvious, which could prove the accuracy of the three characteristic points again.

### 4.3. Verification of Expanded Experimental Data

To verify the accuracy of the NSF method, leave-one-out (LOO) cross validation [26] was performed and compared with FEM simulation. LOO cross validation takes *n* – 1 as the training set, the remaining one is the test set, and then it selects the next one as the test set, and so on to perform *n* cycles. By comparing the experimental data with estimation methods, the relative error *δ* can be calculated by

(14) $$\delta_{i}=\left|\frac{\varepsilon_{i}-\varepsilon_{e}}{\varepsilon_{e}}\times 100\%\right|$$

(15) $$\delta_{s}=\left|\frac{\varepsilon_{s}-\varepsilon_{e}}{\varepsilon_{e}}\times 100\%\right|$$

where *ε_i_*, *ε_s_*, and *ε_e_* are the interpolated, simulated, and experimental strains respectively.

**Figure 8:**
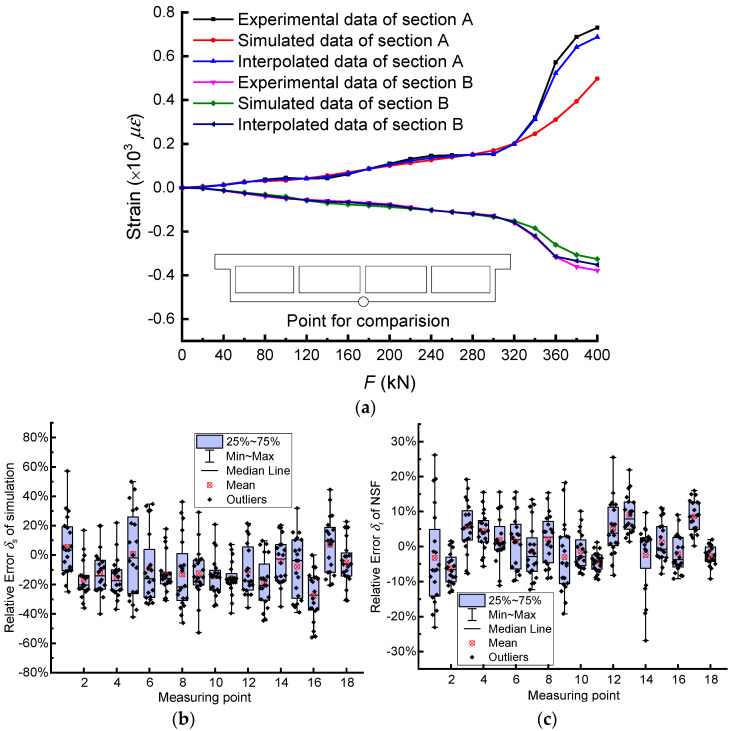
The error comparison between simulated and interpolated data: (**a**) the strain estimation of midpoint by simulation and interpolation, box chart of relative errors of (**b**) simulation, and (**c**) NSF method of section B.

The experimental results, simulation results, and interpolation results based on the NSF method of the strains at the middle of the bottom plate for sections A and B are shown in Figure 8a. It can be observed that the three curves were close before 320 kN. However, when the load exceeded 320 kN, the difference between the simulated data curve and the other two curves increased gradually, while the intervals between interpolated and experimental data were still very small. Furthermore, the relative errors of simulation and the NSF method of different measuring points under all load steps are shown in Figure 8b,c. The maximum and average errors of interpolated data were 26.8% and 0.7%, respectively, for section B. Similarly, the maximum and average errors of simulated data were 57.1% and 10.1%, respectively. It can be seen that the relative error of the NSF method was smaller than that of simulation in all respects. Furthermore, for section A, which is not illustrated here, it showed the same conclusion. By adjusting the model parameters and intrinsic parameters, the simulation results were as close as possible to the experimental ones. However, due to the unknown defects in the structure, construction errors, etc., there were still large differences between the simulation results of strain and the experimental ones at some measuring points. Hence, the NSF method provided a more effective way to expand experimental data and estimate internal forces for the in-depth study of the structural working characteristics.

### 4.4. Analysis of the Expanded Strain and Stress Data

**Figure 9:**
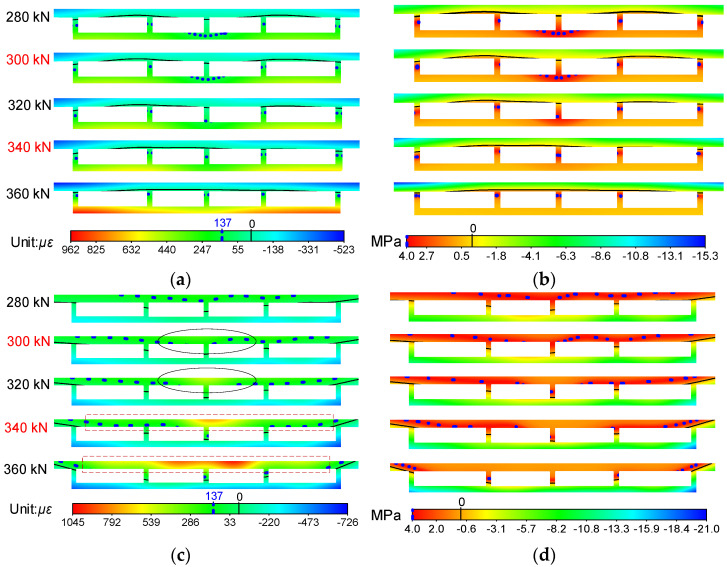
Strain and stress fields of the bridge model constructed by the NSF method: (**a**) strain fields of section A; (**b**) stress fields of section A; (**c**) strain fields of section B; (**d**) stress fields of section B.

In order to compensate to some extent for the difficulties in analyzing the overall working behavior characteristics of the bridge model caused by insufficient experimental data, the NSF method was applied, and the strain and stress fields could be easily obtained. Here, the strain and stress fields of sections A and B from 280 kN to 360 kN are plotted in Figure 9, so that the changing characteristics around characteristic loads *Q* and *H* of the stressing state for the bridge model could be reflected intuitionally from them. The black solid line in the figure represents the point with stress and strain of 0, and the blue dotted line represents the point with maximum tensile stress (137 με) and maximum tensile strain (4 MPa) of concrete.

It can be seen in Figure 9a that the roof and soleplate of section A were respectively subjected to compression and tension, and the line of 0 με was near the bottom of the roof. From 280 kN to load *Q*, except the middle parts, nearly all the strains of the soleplate and the lower half of the webs on both sides were over 137 με (the ultimate tensile strain of the concrete), namely these parts began to crack in different degrees. However, the cracking areas just had a slight increase with the growth of loads, reflecting the accumulation of quantitative instead of qualitative mutations. After that, the entire soleplate exceeded the ultimate tensile strain of the concrete, signifying that the concrete of the entire soleplate basically lost its bearing capacity, and all the tension was borne by the steel bars. The transformation of tension on the soleplate would result in the mutation of the stressing state for the bridge model without doubt, and it may also have contributed to the abrupt change of strain/stress fields for itself or other sections, such as the significant increase and position change of cracking areas, the change of the line of 0 με, etc., which could also reveal the mutation characteristics of the stressing state. The changing characteristics of cracking areas were consistent with the experimental phenomenon; hence, it could be speculated that the whole bridge model entered a failure stressing state. When it surpassed load *H*, the cracking areas spread to nearly all the webs and clung to the line of 0 με, and both ends of the soleplate mutated from green to red, indicating that almost all the tensile zone of the concrete lost its capacity, which would accelerate the failure of the whole bridge model, namely progressive failures. According to the material constitutive relationship, the stress fields can be further obtained, as shown in Figure 9b, and then the mutation characteristics of the stressing state can be analyzed. As for section B shown in Figure 9c,d, the mutation characteristics, including the change of shapes and regions for cracking areas and color gradations, could be obviously seen on the roof of the bridge model at characteristic loads *Q* and *H* as well.

By analyzing the sectional strain/stress fields of the bridge model, the mutation characteristics around the characteristic loads *Q* and *H* and the development trend of strains and stress during the whole loading process could be discovered easily and clearly. Accordingly, the strain/stress fields expanded by the NSF method could reveal the sectional changing characteristics under loading, such as the distribution of the cracking area, the detection of location stress concentration, etc., which could help researchers deeply understand the working behavior of bridges.

**Figure 10:**
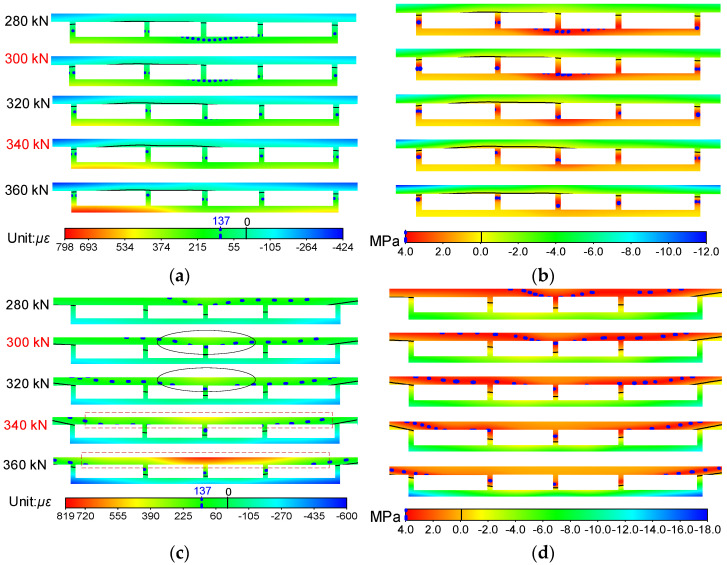
Strain and stress fields of the bridge model obtained by simulation: (**a**) strain fields of section A; (**b**) stress fields of section A; (**c**) strain fields of section B; (**d**) stress fields of section B.

Figure 10 shows the strain and stress fields of sections A and B obtained by simulation, and it can be seen that the changing characteristics of strain and stress fields around 300 kN were not prominent compared with the experimental data. After load *H* (340 kN), the limited strain line of experimental data rose and clung to 0 με, while the line of simulated data rose slowly and had a certain distance from 0 με, signifying that the crack growth speed was slow. Moreover, it could also be observed that the cracking areas did not extend to all the webs for section A and top plate for section B.

### 4.5. Analysis of the Sectional Internal Forces

**Figure 11:**
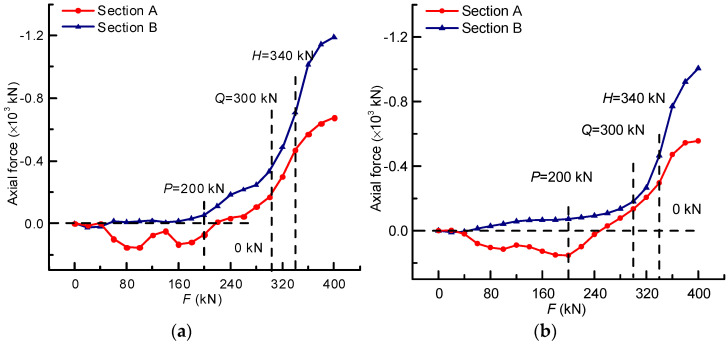
The changing characteristics of the axial force: (**a**) The constructed axial force based on the expanded stress data; (**b**) The axial force of simulated data.

The axial forces of sections A and B could be obtained by Equation (11). It can be seen in Figure 11a that they were quite different in value, while they had nearly the same trend characteristics in the loading process. Before load *P*, they were respectively subjected to adverse axial forces, whereas the values of them were small and had fluctuation, indicating that the bridge model was in the elastic stage with high self-regulation ability. Thereafter, the axial forces of the two sections entered a stable parallel growth stage until load *Q*, and the cracks could be observed clearly on the bottom plate and web, which could be regarded as the steady transformation of the bridge model from the elastic stressing state to the elastic–plastic one. Moreover, the mutation characteristics of the curves after load *Q* could also be discovered, indicating that the stressing state of the bridge model changed from a stable state to an unstable failure one. When it comes to load *H*, the curves of sections A and B bifurcated and never remained parallel, signifying the further aggravation of the bridge model’s failure, namely progressive failures. At the same time, comparing Figure 11a,b, it can be seen that before load *P*, the axial force fluctuation of section A for experimental data was larger than that of the simulated ones. Between loads *P* and *Q*, the axial force growth of section B in Figure 11a was faster than that in Figure 11b. After that, the axial force growth speed of the two figures was almost the same.

**Figure 12:**
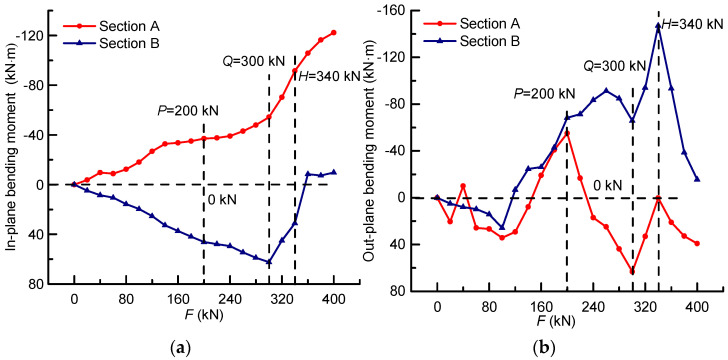
The changing characteristics of the bending moment: (**a**) the constructed in-plane bending moment based on the expanded stress data; (**b**) the constructed out-plane bending moment based on the expanded stress data.

The in-plane and out-plane bending moments of sections A and B could be obtained by Equations (12) and (13), respectively. As for the in-plane bending moments shown in Figure 12a, section B bore sagging bending moments, whereas section A bore the reverse one. Before load *P*, the bending moment of section A fluctuated around 0 kN and that of section B increased gently, which can be considered as the self-regulation results of sectional stress at the elastic stressing state phase. In addition, the mutation characteristic of section A and the maximum in-plane bending moment of section B both occurred at load *Q*, and the developing trend characteristics of the two sections changed from opposite to the same, which can also reveal the mutation of the bridge model’s stressing state. It is remarkable that the bending moment of section B became opposite after load *H*, while that of section A continued to increase sharply at a higher level, reflecting the bridge model’s change of the stressing state and progressive failures.

With regard to straight bridges under the vertical loads, the out-plane bending moments are usually small, which can be ignored in the analysis procedure. Nevertheless, the bridge model is a transverse long cantilever bridge, and in spite of the prestress, the cracks still occur easily, resulting in the nonuniform distribution of stress and internal damage. The influence of nonuniform internal damage would be magnified in the transverse direction, which would cause a certain out-plane bending moment; thus, the change of the out-plane bending moments could embody the nonuniform internal damage to some extent. Correspondingly, the out-plane bending moments were constructed, as shown in Figure 12b, based on the expanded data to investigate the correlation between the change of the structural stressing state and the internal damage. The results show that the bending moments of section A fluctuated around 0 kN by a large margin and the peak ones all occurred at the characteristic loads. As well, the developing trends of sections A and B changed with different stressing state phases, namely they stayed the same at elastic and failure stages and reversed at the elastic–plastic stage, which can also verify the correctness of the stressing state division based on characteristic loads. Therefore, the internal damage is sensitive to the change of stressing state for the bridge model and would change a lot before and after the characteristic loads.

## 5. Discussion

In recent years, it has been widely accepted that the ductility of structures by means of the ductility coefficient can be characterized [27], which is characterized by the deformation of a structure, member, or a certain section of a member [28]. However, the ductility is only reflected by the deformation without characterizing the intrinsic properties, which makes the ductility coefficient scientifically insufficient, and there are many contingencies. Therefore, this paper innovatively divides the ultimate load of the structure by the failure load determined by the structural stressing state theory to represent the ductility, considering the inherent attribute characteristics of the structure, so that the determined ductility will be more convincing and scientific. In addition, many studies have applied the methods mentioned in this paper to various aspects of research. The Mann–Kendall (M–K) criterion is a results-oriented method for trend mutation detection. It does not require that the data must satisfy a specific form of distribution and can allow for missing values [29]. The NSF interpolation method in this study improves the accuracy of the interpolation results by considering the material properties and the actual constraints. In addition, the NSF method combines the spatial interpolation method with numerical simulation, so that this method has some physical significance. It is worth pointing out that the NSF method is currently adopted only for in-plane interpolation, while the application of interpolation in space needs to be further explored. In addition, the structural stressing state theory mentioned in this paper is a theory based on the law of quantitative and qualitative changes, combining classical mechanical theories with empirical and statistical analysis principles to model and analyze the structural response (strains, displacements, etc.), which can reveal the changing characteristics of the structural stressing state and the inherent working mechanism [30]. Although the structural stressing state theory has been successfully applied to conventional structures under conventional loads, its applicability to other forms of structures and loads still needs to be further explored. In a word, the structural stressing state theory can provide a more scientific reference and design basis for structural design.

## 6. Conclusions

This paper investigates the changes of stressing state of the PCTLCC box girder bridge throughout the process from loading to damage by means of structural stressing state theory and the NSF method. The following conclusions are derived from this research:

1. The normalized GSED can effectively model the structural stressing state of the PCTLCC box girder bridge model. Applying the M–K criterion to the $E_{\mathrm{norm}}^{k}$–*F* curve, three characteristic loads, namely elastic–plastic branch load *P* = 200 kN, failure load *Q* = 300 kN, and progressive failure load *H* = 340 kN, are detected to separate three stressing state phases of the bridge model. Then the effectivity and rationality of the M–K criterion are verified through the analyses of measured and expanded data;
2. When the load is less than *P*, the bridge model is in the stable stressing stage. As the load exceeds *P*, the structure starts to enter the elastic–plastic stage, and the strain-*F* curve shows non-linear growth. When the load continues to increase to greater than *Q*, the bridge model enters an unstable stress stage and is unsuitable for further loading, i.e., *Q* is the starting point of the bridge failure process. Furthermore, when the load exceeds *H*, the structure would damage faster than before, indicating the characteristic of progressive failure of the structure;
3. The NSF method provides a more effective way to expand experimental data and estimate internal forces. The maximum and average errors for the NSF method are 26.8% and 0.7%, respectively, but for simulation are 57.1% and 10.1%, meaning that the NSF method has reliable accuracy. Moreover, the strain/stress fields and internal forces constructed by the NSF method and can specifically and intuitively explain the sectional stress developing and damage characteristics in each stressing state phase.

In a word, the structural stressing state theory and NSF method can deeply reveal the working characteristics and the dynamic features of the stressing state of the PCTLCC box girder bridge. Furthermore, this study can provide a new way for the analysis of other types of bridges and has an important reference value for structural design.

## Data Availability

Data are contained within the article.
